# Renin-Angiotensin-Aldosterone System Blockers Are Not Associated With Coronavirus Disease 2019 (COVID-19) Hospitalization: Study of 1,439 UK Biobank Cases

**DOI:** 10.3389/fcvm.2020.00138

**Published:** 2020-07-14

**Authors:** Zahra Raisi-Estabragh, Celeste McCracken, Maddalena Ardissino, Mae S. Bethell, Jackie Cooper, Cyrus Cooper, Nicholas C. Harvey, Steffen E. Petersen

**Affiliations:** ^1^NIHR Barts Biomedical Research Centre, William Harvey Research Institute, Queen Mary University of London, London, United Kingdom; ^2^Barts Heart Centre, St. Bartholomew's Hospital, Barts Health NHS Trust, London, United Kingdom; ^3^Sir Alexander Fleming Building, Imperial College London, London, United Kingdom; ^4^North West Anglia NHS Foundation Trust, Hinchingbrooke Hospital, Huntingdon, United Kingdom; ^5^MRC Lifecourse Epidemiology Unit, University of Southampton, Southampton, United Kingdom; ^6^NIHR Southampton Biomedical Research Centre, University Hospital Southampton NHS Foundation Trust, University of Southampton, Southampton, United Kingdom

**Keywords:** coronavirus disease 2019, UK Biobank, ethnicity, sex, obesity, cardiometabolic disease, Angiotensin Converting Enzyme inhibitors, Angiotensin Receptor Blockers

## Abstract

**Background:** Cardiometabolic morbidity and medications, specifically Angiotensin Converting Enzyme inhibitors (ACEi) and Angiotensin Receptor Blockers (ARBs), have been linked with adverse outcomes from coronavirus disease 2019 (COVID-19). This study aims to investigate, factors associated with COVID-19 positivity in hospital for 1,436 UK Biobank participants; compared with individuals who tested negative, and with the untested, presumed negative, rest of the cohort.

**Methods:** We studied 7,099 participants from the UK Biobank who had been tested for COVID-19 in hospital. We considered the following exposures: age, sex, ethnicity, body mass index (BMI), diabetes, hypertension, hypercholesterolaemia, ACEi/ARB use, prior myocardial infarction (MI), and smoking. We undertook comparisons between (1) COVID-19 positive and COVID-19 negative tested participants; and (2) COVID-19 tested positive and the remaining participants (tested negative plus untested, *n* = 494,838). Logistic regression models were used to investigate univariate and mutually adjusted associations.

**Results:** Among participants tested for COVID-19, Black, Asian, and Minority ethnic (BAME) ethnicity, male sex, and higher BMI were independently associated with a positive result. BAME ethnicity, male sex, greater BMI, diabetes, hypertension, and smoking were independently associated with COVID-19 positivity compared to the remaining cohort (test negatives plus untested). However, similar associations were observed when comparing those who tested negative for COVID-19 with the untested cohort; suggesting that these factors associate with general hospitalization rather than specifically with COVID-19.

**Conclusions:** Among participants tested for COVID-19 with presumed moderate to severe symptoms in a hospital setting, BAME ethnicity, male sex, and higher BMI are associated with a positive result. Other cardiometabolic morbidities confer increased risk of hospitalization, without specificity for COVID-19. ACE/ARB use did not associate with COVID-19 status.

## Introduction

Coronavirus disease 2019 (COVID-19), the clinical illness caused by the severe acute respiratory syndrome coronavirus 2 (SARS-CoV-2), has reached pandemic levels. There has been growing recognition that patients with underlying cardiometabolic morbidities may be suffering higher rates of infection and a more severe disease course than the general population (1–3). Debate has ensued regarding whether these observations relate to the conditions themselves or the medications with which they are treated. In particular, some have suggested a mechanistic role for Angiotensin Converting Enzyme inhibitors (ACEi) or Angiotensin Receptor Blockers (ARBs) (4). However, recent reports have not produced convincing evidence for the specific association of ACEi/ARBs with poorer outcomes (4–6). Cardiometabolic diseases are common and ACEi/ARBs are used by many vulnerable patients. It is therefore important to better understand the augmented risk associated with cardiometabolic factors and ACEi/ARB use with COVID-19, to inform clinical practice and guidance to patients.

The UK Biobank (UKB) is a large cohort study comprising data from over 500,000 participants from across the UK, characterized in detail at baseline (2006–2010), and with linkages to Hospital Episode Statistic (HES) data. In response to the COVID-19 pandemic, the UKB facilitated rapid release of COVID-19 testing data for its participants through linkage with Public Health England (7), providing a unique opportunity to study the effects of many well-defined exposures on COVID-19 status.

The aim of this study is to investigate the association of demographic factors (age, sex, ethnicity), cardiometabolic profile [body mass index (BMI), diabetes, hypertension, hypercholesterolaemia, prior myocardial infarction (MI), smoking], and ACEi/ARB use with COVID-19 positivity in hospital using data from UKB.

## Methods

### Setting and Study Population

UKB is a prospective cohort study including over 500,000 participants from across the UK. Individuals aged 40–69 years old identified via National Health Service (NHS) registers were recruited over a 4-year period between 2006 and 2010. Participants underwent detailed baseline assessment including characterization of socio-demographics, lifestyle, medical history, and a series of physical measures. The protocol is publicly available (8). Linkages with HES data permit longitudinal tracking of health outcomes for all participants with conditions recorded according to international classification of disease (ICD) codes. In addition, UKB has produced algorithmically defined outcome data for incidence of key illness, such as MI, through integration of data from multiple sources (9). The latest data release (24th June 2020) includes test results from 16th March to 14th June. In the UK, until the 18th of May 2020, testing was almost entirely limited to hospital settings, after this date, testing was extended to the community. Therefore, we consider a positive test performed up to the 18th of May as indicative of hospitalization, beyond this date we required explicitly labeling of the sample as “inpatient.” Testing was based on a real-time polymerase chain reaction (RT-PCR) assay antigen test; for most participants the sample tested was from combined nose and throat swab; for patients in intensive care lower respiratory samples may have been used. Thus, we defined a cohort of participants who were tested for SARS-CoV-2 whilst admitted to hospital, and therefore are likely to have a relatively severe presentation.

### Statistical Analysis

**Figure 1:**
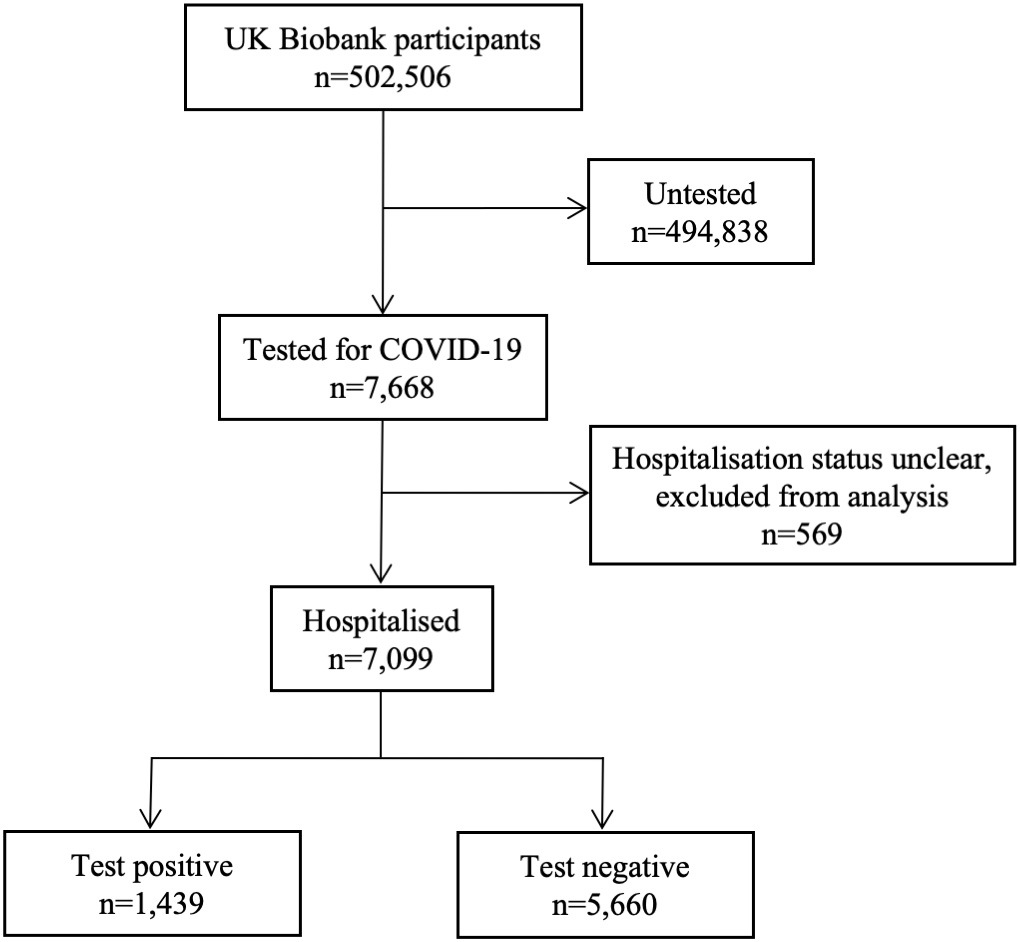
Summary of COVID-19 testing and results for UK Biobank participants. Data includes COVID-19 test results from 16/03/2020 to 14/06/2020. During this time period, 7,688 participants, of the whole UK Biobank cohort (*n* = 502,506) have been tested for COVID-19. 7,099 were likely in a hospital setting, of whom 1,439 participants had a positive result and 5,660 tested negative. The remaining participants (*n* = 494,838) have not been tested.

Statistical analysis was performed using R Version 3.6.2 (10), and RStudio Version 1.2.5019 (11). We considered the following exposures: age, sex, ethnicity, body mass index (BMI), diabetes, hypertension, high cholesterol, ACEi/ARB use, prevalent MI, and smoking. The cardiometabolic and demographic factors were selected based on existing reports of their potential association with COVID-19 outcomes (3, 12, 13). ACEi/ARBs were considered due to reports of potential mechanistic role of these medications in the clinical course of COVID-19 (4). We used age, sex, and ethnicity (White vs. BAME) as recorded at baseline. BMI was calculated from height and weight recorded at baseline. Smoking status was based on self-report. Hypertension, diabetes, and hypercholesterolaemia were defined through cross-checking across self-report and HES data. A list of ICD codes used is available in Supplementary Table 1. Information on prior MI was obtained from the UKB algorithmically defined health outcomes. ACEi/ARB use was determined from self-report (Supplementary Table 2). We considered the effect of ACEi and ARBs both separately and as an aggregate variable. We created three cohorts: test positives, test negatives, and the untested cohort (Figure 1). Individuals who were tested, but with unclear hospitalization status were excluded from the analysis. We firstly compared the COVID-19 test positive cohort with the combined cohort of test negatives and the untested UKB population. In order to investigate possible bias relating to hospitalization status, we also considered the importance of these exposure variables in two further comparisons: test positives vs. test negatives and test negatives vs. untested population. We used logistic regression models to elucidate univariate and then multivariate associations. There was no evidence of multicollinearity with variance inflation factor (VIF) <2.0 for all covariates. As the observed association with ethnicity was strong, we tested for potential interaction effects between ethnicity and all tested covariates in multivariate models. We present odds ratio (OR) for each exposure with the corresponding 95% confidence interval (CI) and *p*-value. Given the low background prevalence of COVID-19 positivity, the odds ratios can be interpreted as relative risks.

## Results

### Baseline Characteristics

Of the 7,668 UKB participants tested for COVID-19, 7,099 were likely in a hospital setting and are included in this analysis (Table 1, Figure 1), of these 1,439 tested positive and 5,660 tested negative. There was no record of testing for the remainder of the UKB cohort (*n* = 494,838) (Figure 1).

**Table 1:** Baseline participant characteristics.

	**COVID-19 tested (*n* = 7,099)**	**COVID-19 positive (*n* = 1,439)**	**COVID-19 negative (*n* = 5,660)**	**Untested population (*n* = 494,838)**
Sex (Male)	3,525 (49.7%)	761 (52.9%)	2,764 (48.8%)	225,352 (45.5%)
Age^*^	69.11 (±8.65)	68.22 (±9.19)	69.34 (±8.49)	68.24 (±8.10)
White ethnicity	6,498 (91.5%)	1,242 (86.3%)	5,256 (92.9%)	465,681 (94.1%)
BAME ethnicity	562 (7.9%)	185 (12.9%)	377 (6.7%)	26,429 (5.3%)
BMI (kg/m^2^)	27.66 [24.78, 31.13]	27.97 [25.18, 31.50]	27.58 [24.69, 31.02]	26.7 [±24.13, 29.89]
Smoking^**^	3,663 (51.6%)	732 (50.9%)	2,931 (51.8%)	221,478 (44.8%)
Prior MI	557 (7.8%)	103 (7.2%)	454 (8.0%)	20,227 (4.1%)
Diabetes	1,029 (14.5%)	241 (16.7%)	788 (13.9%)	38,046 (7.7%)
Hypertension	3,338 (47.0%)	676 (47.0%)	2,662 (47.0%)	171,415 (34.6%)
High cholesterol	2,388 (33.6%)	477 (33.1%)	1,911 (33.8%)	115,133 (23.3%)
ACEi^†^	1,117 (15.7%)	227 (15.8%)	890 (15.7%)	50,635 (10.2%)
ARB^†^	418 (5.9%)	87 (6.0%)	331 (5.8%)	20,416 (4.1%)

*Data are n (%), mean (standard deviation), or median [interquartile range]. COVID-19 data includes test results from 16/03/2020 to 14/06/2020 from hospital settings*.

* *We report age of participants as of 01/04/2020*.

** *Smoking includes current and previous smoking*.

† *ACEi/ARB use is defined as a binary measure, defined as true if record of any of medications in Supplementary Table 2. ACEi, Angiotensin Converting Enzyme inhibitor; ARB, Angiotensin Receptor Blocker; BAME, Black, Asian, and Minority ethnic; BMI, body mass index; COVID-19, coronavirus 2019*.

In comparison to the untested cohort, the COVID-19 positive cohort were predominantly male (52.9% vs. 45.5%), had a greater proportion of BAME individuals (12.9% vs. 5.3%), and an all-round poorer cardiometabolic profile, with higher BMI, higher rates of smoking, prior MI, diabetes, hypertension, and high cholesterol; they also reported greater use of ACEi/ARB agents (21.8% vs. 14.3%). However, comparing the COVID-19 positive cohort with the tested negative cohort (*n* = 5,660), the differences were much less pronounced, as the test negative cohort also had a globally poorer cardiometabolic profile than the untested population.

### Association of Exposures With COVID Status

#### COVID-19 Positive vs. Not COVID-19 Positive (Tested Negative Cohort Plus Untested Cohort)

**Figure 2:**
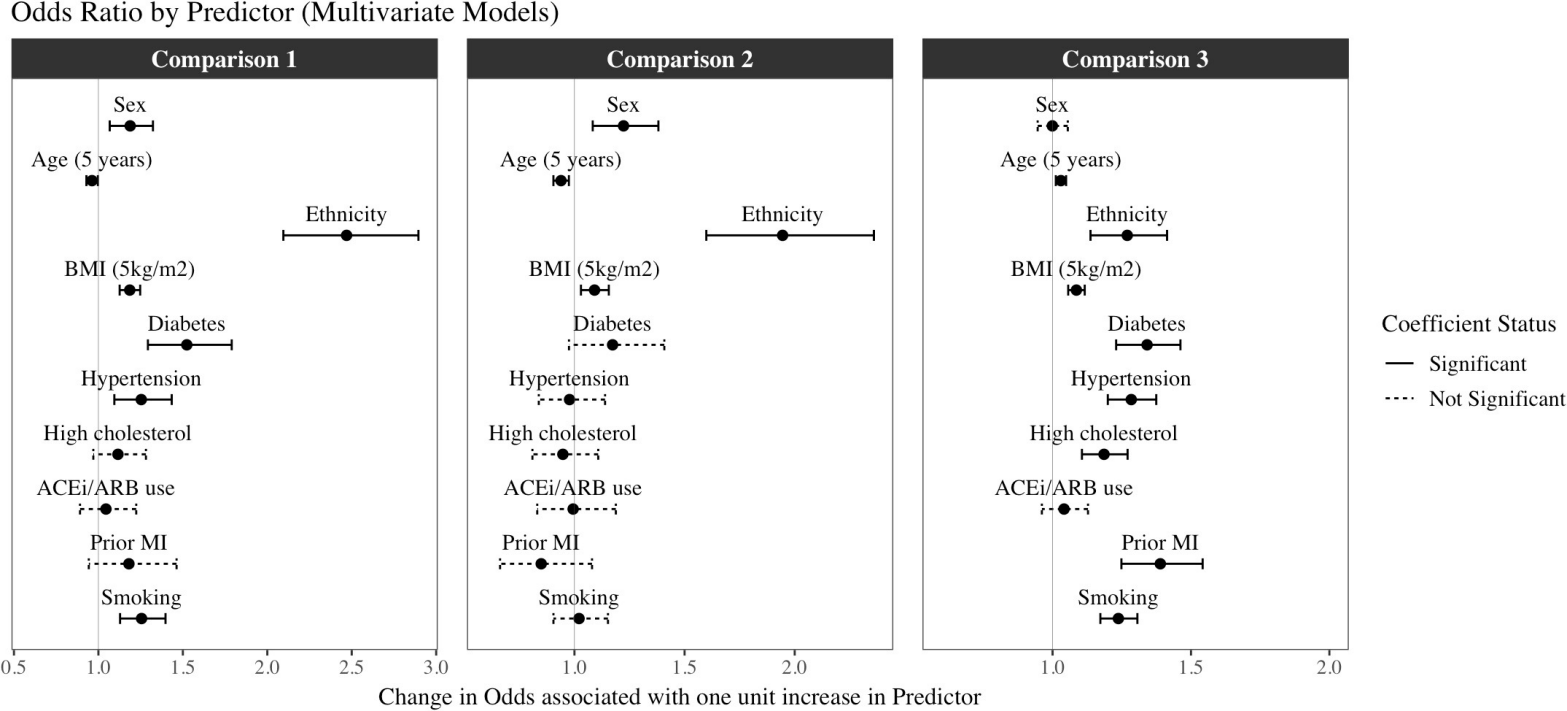
Odds Ratios and 95% confidence intervals for each exposure from the multivariate logistic regression models in the three different comparisons*. *Comparison 1: COVID-19 positive (*n* = 1,439) vs. not COVID-19 positive (tested negative plus untested cohort) (*n* = 494,838); Comparison 2: COVID-19 positive (*n* = 1,439) vs. COVID-19 test negative (*n* = 5,660); Comparison 3: COVID-19 test negative (*n* = 5,660) vs. untested population (*n* = 494,838). Results are odds ratios with 95% confidence intervals. Dashed lines represent non-significant and solid lines statistically significant results, with threshold at *p* < 0.05.

We first tested whether there were univariate associations between exposures and COVID-19 positives (*n* = 1,439) vs. not COVID-19 positives (including tested negative and untested cohort, *n* = 500,498). Univariate associations were significant for all covariates considered, except age. In multivariate models, the independent predictors of COVID-19 positivity were younger age, male sex, BAME ethnicity, greater BMI, diabetes, hypertension, and smoking (Table 2, Figure 2: Comparison 1).

**Table 2:** Odds Ratios, 95% confidence intervals, and p-values for each exposure from univariate and multivariate logistic regression models in the three defined comparisons^**^.

	**Comparison 1**		**Comparison 2**		**Comparison 3**	
**Predictors**	**Univariate Models**	**Multivariate Model**	**Univariate Models**	**Multivariate Model**	**Univariate Models**	**Multivariate Model**
Male sex	1.34^*^ [1.21, 1.49]	1.19^*^ [1.07, 1.32]	1.18^*^ [1.05, 1.32]	1.22^*^ [1.08, 1.38]	1.14^*^ [1.08, 1.20]	1.00 [0.95, 1.06]
	3.07 × 10^−8^	0.0017	0.0061	0.0012	7.68 × 10^−7^	0.9759
Age (per 5 years)	1.00 [0.97, 1.03]	0.96^*^ [0.93, 1.00]	0.93^*^ [0.90, 0.96]	0.94^*^ [0.90, 0.97]	1.09^*^ [1.07, 1.11]	1.03^*^ [1.01, 1.05]
	0.8620	0.0316	1.17 × 10^−5^	9.64 × 10^−4^	5.81 × 10^−24^	0.0013
BAME ethnicity	2.62^*^ [2.23, 3.05]	2.47^*^ [2.10, 2.89]	2.08^*^ [1.72, 2.50]	1.95^*^ [1.60, 2.36]	1.26^*^ [1.14, 1.40]	1.27^*^ [1.14, 1.41]
	4.58 × 10^−34^	5.58 × 10^−28^	1.59 × 10^−14^	2.07 × 10^−11^	1.29 × 10^−5^	1.70 × 10^−5^
BMI (per 5kg/m2)	1.30^*^ [1.24, 1.36]	1.19^*^ [1.13, 1.25]	1.10^*^ [1.04, 1.16]	1.09^*^ [1.03, 1.16]	1.19^*^ [1.16, 1.22]	1.09^*^ [1.06, 1.12]
	2.19 × 10^−29^	7.63 × 10^−11^	3.62 × 10–4	0.0031	4.47 × 10^−42^	3.78 × 10^−9^
Diabetes	2.39^*^ [2.08, 2.74]	1.52^*^ [1.29, 1.79]	1.24^*^ [1.06, 1.45]	1.17 [0.98, 1.41]	1.94^*^ [1.80, 2.09]	1.34^*^ [1.23, 1.46]
	7.39 × 10^−35^	3.72 × 10^−7^	0.0066	0.0882	1.05 × 10^−65^	2.80 × 10^−11^
Hypertension	1.66^*^ [1.50, 1.84]	1.25^*^ [1.09, 1.43]	1.00 [0.89, 1.12]	0.98 [0.84, 1.14]	1.68^*^ [1.59, 1.77]	1.28^*^ [1.20, 1.37]
	8.27 × 10^−22^	0.0010	0.9704	0.7727	1.27 × 10^−82^	5.90 × 10^−13^
High cholesterol	1.62^*^ [1.45, 1.81]	1.12 [0.97, 1.28]	0.97 [0.86, 1.10]	0.95 [0.81, 1.11]	1.68^*^ [1.59, 1.78]	1.19^*^ [1.11, 1.27]
	5.20 × 10^−18^	0.1234	0.6592	0.5006	3.31 × 10^−75^	1.52 × 10^−6^
ACEi/ARB	1.65^*^ [1.45, 1.87]	1.04 [0.89, 1.22]	1.01 [0.88, 1.17]	0.99 [0.83, 1.19]	1.64^*^ [1.54, 1.75]	1.04 [0.96, 1.13]
	7.54 × 10^−15^	0.5885	0.8563	0.9468	2.31 × 10^−51^	0.3193
Prior MI	1.79^*^ [1.45, 2.17]	1.18 [0.94, 1.46]	0.88 [0.70, 1.10]	0.85 [0.66, 1.08]	2.05^*^ [1.85, 2.25]	1.39^*^ [1.25, 1.54]
	1.41 × 10^−8^	0.1377	0.2770	0.1893	1.70 × 10^−47^	1.02 × 10^−9^
Smoking	1.27^*^ [1.15, 1.41]	1.26^*^ [1.13, 1.40]	0.96 [0.86, 1.08]	1.02 [0.90, 1.15]	1.33^*^ [1.26, 1.40]	1.24^*^ [1.17, 1.31]
	4.58 × 10^−6^	3.02 × 10^−5^	0.5348	0.7369	5.91 × 10^−26^	9.40 × 10^−15^

** *Comparison 1: COVID-19 positive (n = 1,439) vs. not COVID-19 positive (tested negative plus untested cohort) (n = 494,838); Comparison 2: COVID-19 positive (n = 1,439) vs. COVID-19 test negative (n = 5,660); Comparison 3: COVID-19 test negative (n = 5,660) vs. untested population (n = 494,838). Results are odds ratio, 95% confidence interval, and p-value (from top to bottom) for each exposure. For continuous variables (age, BMI) coefficients refer to the effect on odds of the outcome per five unit increase in the exposures, i.e., 5-year increase in age and 5 kg/m^2^ increase in BMI. The remaining exposures are set as binary measures with results showing effect of change from non-disease to disease states, male sex vs. female sex, BAME ethnicity vs. White ethnicity; smoking history (current/previous) vs. never smoked; ACEi/ARB use vs. no ACEi/ARB use on odds of the outcome*.

* *Indicates p < 0.05. ACEi, Angiotensin Converting Enzyme inhibitor; ARB, Angiotensin Receptor Blocker; BMI, body mass index; coronavirus 2019: COVID-19; BAME, Black, Asian, and Minority ethnic; MI, myocardial infarction*.

#### COVID-19 Positive vs. COVID-19 Tested Negative

We next considered associations between exposures and COVID-19 positives (*n* = 1,439) vs. tested negative cohort (*n* = 5,660). Within this sample, the univariate predictors of positivity were male sex, younger age, BAME ethnicity, greater BMI, and diabetes. These variables, with the exception of diabetes, remained statistically significant in the multivariate model with mutual adjustment for all other covariates (Table 2, Figure 2). The greatest magnitude of effect related to ethnicity; BAME individuals had almost twice the likelihood of a COVID-19 positive result compared to White ethnicities in the fully adjusted models [OR 1.95, 95% CI (1.60, 2.36)]. There was no evidence of interaction effect with ethnicity and any of the other covariates (Supplementary Table 3). Compared with women, men had 22% greater odds of a COVID-19 positive test [OR 1.22, 95% CI (1.08, 1.38)]. For every 5 kg/m^2^ increase of BMI, there was 9% greater odds of COVID-19 positive status (Table 2, Figure 2: Comparison 2). There was a negative association with age, this may reflect older age of participants admitted to hospital for reasons other than COVID-19; alternatively, it may be an artifact of the data related to the narrow age range in the sample. Notably, there was no significant association between ACEi/ARB use and COVID-19 status, which was consistent when testing effect of ACEi and ARBs separately (Supplementary Table 4).

#### COVID-19 Tested Negatives vs. Untested Population

Finally, we investigated associations between the exposures with a negative test (*n* = 5,660) vs. untested UKB population (*n* = 494,838). There were significant univariate associations for all covariates considered. In the multivariate model, BAME ethnicity, older age, higher BMI, diabetes, hypertension, high cholesterol, previous MI, and smoking were significant predictors of a having a negative test, and therefore of presenting to hospital, perhaps with respiratory symptoms, compared to not being tested (Table 2, Figure 2: Comparison 3).

## Discussion

### Summary of Findings

In this analysis of 7,099 UKB participants tested for COVID-19 in a hospital setting, BAME ethnicity, younger age, male sex, greater BMI, diabetes, hypertension, and smoking were independently associated with COVID-19 positive test in comparison to the rest of the cohort (tested negatives plus untested). However, within the tested sample, a positive result was more likely for men, BAME individuals, younger ages, and with greater BMI. Indeed, when compared with the background population, the pattern of associations between exposures and COVID-19 positive was similar to that for COVID-19 test negative. These findings suggest that BAME ethnicity, male sex, and higher BMI have specific relevance to COVID-19, whilst the other exposure associations between COVID-19 positive and the remainder of the population reflect morbidities associated with general requirement for hospitalization, without specificity to COVID-19. Furthermore, as testing was in a hospital setting, these associations relate specifically to the more severe end of the COVID-19 manifestations requiring hospitalization. Notably, ACEi/ARB usage was not associated with COVID-19 status.

### Comparison With Existing Literature

With the rapid global spread of COVID-19, understanding the determinants of infection risk and severity is a priority. Differences in ethnic background are known to contribute to differences in patterns of a number of diseases, including influenza (14), due to different genetic susceptibilities and environmental exposures (15). In the UK, national audit data demonstrates as many as one-third of COVID-19 patients admitted to intensive care are from BAME backgrounds; a rate which is disproportionate to their representation among the general UK population (16). In our study, BAME ethnicity had specific association with higher risk of COVID-19 positive status that appeared independent from often-quoted confounders of cardiovascular and metabolic morbidity that are known to be higher in prevalence in BAME cohorts (17). Having accounted for cardiometabolic morbidity, the possible explanations for this association remain numerous (18), gravitating around both genetic and social factors; behavioral, cultural, and socioeconomic differences, including health-seeking behavior and intergenerational cohabitation are all likely to play a role in the strong disparity observed in our study, providing key targets for both further research and public health policy. Initial studies, demonstrate complex interplay of biological and socio-economic factors and highlight need for urgent research in this area (19).

Since the first reports emerging from China at the beginning of the outbreak, it has been widely recognized that males suffer higher rates of infection and poorer outcomes compared to females; with reported distributions of approximately three-fifths men and two-fifths women (20, 21). The reasons for this are unclear. Animal studies demonstrate, that in mice infected with SARS-CoV, estrogen-deplete status either due to male gender or ovariectomy is associated with higher risk of acute respiratory distress syndrome (ARDS), indicating a possible protective role of estrogen signaling (22). Men are known to have higher burden of cardiovascular disease than women up to the perimenopausal years; and thus, lower cardiometabolic morbidity among women in the younger cohort has been postulated to contribute to better outcomes. However, we demonstrate that in our study population, the association between male sex and higher infection rates was independent of cardiometabolic disease. Furthermore, male sex appears significant in our sample comprising an older cohort with almost all women being post-menopause, indicating that sex-differential disparities in COVID-19 disease severity relate to factors other than immediate-term estrogen exposure. Thus, our findings suggest that the higher risk of COVID-19 in men is not sufficiently explained by the estrogen pathway or greater burden of cardiometabolic disease.

Obesity is a global health issue, rising in prevalence and public health burden in both developed and developing countries. Patients who suffer from obesity are known to be at increased risk of a number of conditions, including cardiometabolic and respiratory disease, contributing to a poor physiological reserve. It is already known that patients with obesity have worse outcomes from influenza infection (23, 24). With the wealth of emerging research on COVID-19, concern has grown over the association between obesity and poor outcomes of infection (25); with studies consistently demonstrating higher rates of critical or intensive care requirement among individuals with higher BMI (26–28). Similar to ethnicity, the relationship between obesity and severe infection must be isolated from the confounding of obesity-related comorbidity. In our study, we demonstrate the distinct role of obesity from that of associated cardiometabolic diseases; with the major finding that obesity, and not its comorbidities, had independent and specific association with COVID-19 positivity. This is of important relevance, as mechanistic understanding of the reason behind this association may provide therapeutic insight. For example, obesity enhances risk of thrombosis, which has been a recent focus of interest given concern over a possible association between COVID-19 and prothrombotic intravascular coagulation (29). The results of our study provide useful information for risk stratification of patients, highlight important avenues for further research, and emphasize the public health-level importance of continued targeting of obesity.

Several reports hypothesize potential mechanistic links between ACEi/ARB usage and adverse outcomes from COVID-19 (4). SARS-CoV-2 has been shown to exhibit specific tropism for the angiotensin-converting enzyme 2 (ACE2) receptor; by which means it enters the cells and establishes itself in the host (30). The expression of ACE2 receptors in epithelial cells of the lung, intestine, kidney and endothelium may be increased in those treated with ACEi/ARBs, thereby facilitating entry and multisystem manifestations of COVID-19 (31, 32). The relationship between COVID19 infection risk and use of ACEi/ARBs has been a matter of debate since the early days of the outbreak, but recent studies have revealed a lack of independent association when morbidity variables, including atherosclerotic cardiovascular disease, heart failure and cardiometabolic diseases such as diabetes and hypertension were accounted for (4, 5). Furthermore, a recent study from Spain demonstrates no association between ACEi/ARB use and COVID-19 mortality or requirement for intensive care (33). Findings from our sample are consistent with these reports, demonstrating univariate association with ACEi/ARB use which becomes non-significant after adjustment for cardiometabolic and demographic factors.

### Strengths and Limitations

UKB is a comprehensive data source, incorporating a large sample with linkages to prospectively tracked health outcomes recorded in a standardized manner using ICD codes, enabling reliable and up-to-date definition of morbidities. The rapid release of COVID-19 testing data provides a huge opportunity to examine association of a large number of exposures with COVID-19 status and outcomes. Due to the observational study design, we cannot comment on causal relationships from the results, however, the prospective nature of the study ensures confident temporal separation of exposure and outcome. Whilst analyses using the whole UK Biobank cohort of over 500,000 people may detect very small associations which are unlikely to be clinically significant, we studied a subset of much more modest sample size, with exposures and covariates chosen on the basis of prior literature and biological plausibility with the magnitude of relationships observed likely to be clinically meaningful. Further research in different cohorts would be helpful in better understanding the impact of the exposures studied. Whilst we can be reasonably confident about hospitalization status of the tested cohort in this study, there is uncertainty about the degree of symptoms. We acknowledge that there are local variations in testing approaches and that conclusions regarding disease severity drawn from hospitalization status alone have limitations. Studies in cohorts with more granular outcome data are needed. Furthermore, our results cannot be generalizable to asymptomatic or mildly symptomatic patients.

## Conclusions

This work highlights specific associations of BAME ethnicity, male sex, and higher BMI with COVID-19 positive status, which were independent of other demographic or cardiometabolic factors. More detailed characterization of these associations in larger and more diverse cohorts is warranted, particularly with regards ethnicity. Investigation of potential biological pathways underlying these observed associations may provide insight into the mechanisms by which SARS-CoV-2 causes disease enabling more informed pursuit of potential therapeutic targets.

## Data Availability Statement

This study was performed using data from the UK Biobank under access application 2964. The UK Biobank is an open access research resource with data available on request to all bone fide researchers through the UK Biobank website: http://www.ukbiobank.ac.uk.

## Ethics Statement

This study was covered by the ethics approval for UKB studies from the NHS National Research Ethics Service on 17th June 2011 (Ref 11/NW/0382) and extended on 10th May 2016 (Ref 16/NW/0274). The patients/participants provided their written informed consent to participate in this study.

## Author Contributions

This study was conceived by ZR-E, SP, and NH. The manuscript was written by ZR-E, MA, and MB. SP, NH, and CC advised on revisions of the manuscript. CM led on and conducted the statistical analysis. JC provided statistical advice. All co-authors read and approved the manuscript.

## Conflict of Interest

The authors declare that the research was conducted in the absence of any commercial or financial relationships that could be construed as a potential conflict of interest.
