# Supplementary material for: Renin-Angiotensin-Aldosterone System Blockers Are Not Associated With Coronavirus Disease 2019 (COVID-19) Hospitalization: Study of 1,439 UK Biobank Cases
Source: Front Cardiovasc Med. 2020 Jul 14;7:138. doi: 10.3389/fcvm.2020.00138 (PMC7381180; doi:10.3389/fcvm.2020.00138)
Supplement: Supplementary file 2 [file Table_2.DOCX]

**Supplementary Table 2. Preparations of angiotensin converting enzyme inhibitor and angiotensin II receptor blockers considered in analysis**

| acepril 12.5mg tablet |
| --- |
| amias 2mg tablet |
| angiotensin ii receptor antagonist |
| aprovel 75mg tablet |
| candesartan cilexetil |
| capoten 12.5mg tablet |
| captopril |
| captopril+hydrochlorothiazide 25mg/12.5mg tablet |
| carace 2.5mg tablet |
| cilazapril |
| co-diovan 80mg/12.5mg tablet |
| coaprovel 150mg/12.5mg tablet |
| coversyl 2mg tablet |
| cozaar half strength 25mg tablet |
| cozaar-comp 50mg/12.5mg tablet |
| diovan 40mg capsule |
| enalapril |
| enalapril maleate+hydrochlorothiazide 20mg/12.5mg tablet |
| eprosartan |
| fosinopril |
| gopten 500micrograms capsule |
| innovace 2.5mg tablet |
| innozide tablet |
| irbesartan |
| irbesartan+hydrochlorothiazide 150mg/12.5mg tablet |
| lisinopril |
| losartan |
| losartan potassium+hydrochlorothiazide 50mg/12.5mg tablet |
| moexipril |
| perindopril |
| quinapril |
| ramipril |
| telmisartan |
| teveten 300mg tablet |
| trandolapril |
| valsartan |
| valsartan+hydrochlorothiazide 80mg/12.5mg tablet |
| vascace 250micrograms tablet |
| zestril 2.5mg tablet |
