# Supplementary material for: Renin-Angiotensin-Aldosterone System Blockers Are Not Associated With Coronavirus Disease 2019 (COVID-19) Hospitalization: Study of 1,439 UK Biobank Cases
Source: Front Cardiovasc Med. 2020 Jul 14;7:138. doi: 10.3389/fcvm.2020.00138 (PMC7381180; doi:10.3389/fcvm.2020.00138)
Supplement: Supplementary file 3 [file Table_3.DOCX]

**Supplementary Table 3. Odds ratios, 95% confidence interval, and p-values for the interaction terms between ethnicity and each predictor in multivariate models with mutual adjustment for ethnicity, age, sex, BMI, diabetes, hypertension, high cholesterol, ACEi/ARB use, previous MI, and smoking. Modelling is within the tested cohort (test positives vs test negatives).**

| Interaction term | Odds ratio [95% confidence interval]  p-value |
| --- | --- |
| BAME ethnicity and Age (5 years) | 1.00 [0.89, 1.12] |
|  | 0.9824 |
| BAME ethnicity and Male sex | 1.17 [0.80, 1.72] |
|  | 0.4075 |
| BAME ethnicity and BMI (per 5kg/m^2^) | 1.10 [0.93, 1.30] |
|  | 0.2830 |
| BAME ethnicity and Diabetes | 0.67 [0.42, 1.05] |
|  | 0.0861 |
| BAME ethnicity and Hypertension | 1.25 [0.86, 1.83] |
|  | 0.2472 |
| BAME ethnicity and High cholesterol | 0.90 [0.60, 1.34] |
|  | 0.5993 |
| BAME ethnicity and ACEi/ARB use | 1.20 [0.76, 1.87] |
|  | 0.4347 |
| BAME ethnicity and Previous MI | 1.17 [0.52, 2.51] |
|  | 0.7024 |
| BAME ethnicity and Smoker Status | 0.91 [0.61, 1.36] |
|  | 0.6616 |

**Supplementary Table 3 footnote:** ACEi: Angiotensin Converting Enzyme inhibitor; ARB: Angiotensin Receptor Blocker; BMI: body mass index; coronavirus 2019: COVID-19; BAME: Black, Asian, and Minority ethnic; MI: myocardial infarction.
