# Supplementary material for: Renin-Angiotensin-Aldosterone System Blockers Are Not Associated With Coronavirus Disease 2019 (COVID-19) Hospitalization: Study of 1,439 UK Biobank Cases
Source: Front Cardiovasc Med. 2020 Jul 14;7:138. doi: 10.3389/fcvm.2020.00138 (PMC7381180; doi:10.3389/fcvm.2020.00138)
Supplement: Supplementary file 4 [file Table_4.DOCX]

**Supplemental Table 4. Odds Ratios, 95% confidence intervals, and p-values from univariate and multivariate logistic regression models in the three defined comparisons**

|  | Comparison 1 | | Comparison 2 | | Comparison 3 | |
| --- | --- | --- | --- | --- | --- | --- |
| Predictors | Univariate Models | Multivariate Model | Univariate Models | Multivariate Model | Univariate Models | Multivariate Model |
| Male sex | 1.34* [1.21, 1.49] | 1.19* [1.07, 1.32] | 1.18* [1.05, 1.32] | 1.22* [1.08, 1.38] | 1.14* [1.08, 1.20] | 1.00 [0.94, 1.05] |
|  | 3.07x10^-8^ | 0.0018 | 0.0061 | 0.0012 | 7.68x10^-7^ | 0.9379 |
| Age (per 5 years) | 1.00 [0.97, 1.03] | 0.96* [0.93, 1.00] | 0.93* [0.90, 0.96] | 0.94* [0.90, 0.97] | 1.09* [1.07, 1.11] | 1.03* [1.01, 1.05] |
|  | 0.8620 | 0.0336 | 1.17x10^-5^ | 9.80x10^-4^ | 5.81x10^-24^ | 0.0012 |
| BAME ethnicity | 2.62* [2.23, 3.05] | 2.47* [2.10, 2.90] | 2.08* [1.72, 2.50] | 1.95* [1.60, 2.36] | 1.26* [1.14, 1.40] | 1.27* [1.14, 1.42] |
|  | 4.58x10^-34^ | 4.60x10^-28^ | 1.59x10^-14^ | 1.99x10^-11^ | 1.29x10^-5^ | 1.48x10^-5^ |
| BMI (per 5kg/m2) | 1.30* [1.24, 1.36] | 1.19* [1.13, 1.25] | 1.10* [1.04, 1.16] | 1.09* [1.03, 1.16] | 1.19* [1.16, 1.22] | 1.09* [1.06, 1.12] |
|  | 2.19x10^-29^ | 6.55x10^-11^ | 3.62x10^-4^ | 0.0031 | 4.47x10^-42^ | 3.03x10^-9^ |
| Diabetes | 2.39* [2.08, 2.74] | 1.52* [1.29, 1.79] | 1.24* [1.06, 1.45] | 1.17 [0.98, 1.41] | 1.94* [1.80, 2.09] | 1.34* [1.23, 1.46] |
|  | 7.39x10^-35^ | 3.52x10^-7^ | 0.0066 | 0.0867 | 1.05x10^-65^ | 2.75x10^-11^ |
| Hypertension | 1.66* [1.50, 1.84] | 1.26* [1.10, 1.44] | 1.00 [0.89, 1.12] | 0.98 [0.84, 1.14] | 1.68* [1.59, 1.77] | 1.29* [1.20, 1.38] |
|  | 8.27x10^-22^ | 8.46x10^-4^ | 0.9704 | 0.7894 | 1.27x10^-82^ | 3.85x10^-13^ |
| High cholesterol | 1.62* [1.45, 1.81] | 1.12 [0.97, 1.28] | 0.97 [0.86, 1.10] | 0.95 [0.81, 1.11] | 1.68* [1.59, 1.78] | 1.19* [1.11, 1.27] |
|  | 5.20x10^-18^ | 0.1210 | 0.6592 | 0.5067 | 3.31x10^-75^ | 1.55x10^-6^ |
| ACEi | 1.63* [1.41, 1.88] | 1.06 [0.90, 1.26] | 1.00 [0.85, 1.17] | 1.00 [0.82, 1.21] | 1.64* [1.52, 1.76] | 1.06 [0.98, 1.16] |
|  | 1.39x10^-11^ | 0.4702 | 0.9626 | 0.9972 | 7.38x10^-41^ | 0.1632 |
| ARB | 1.49* [1.19, 1.84] | 0.96 [0.75, 1.21] | 1.04 [0.81, 1.32] | 0.96 [0.73, 1.25] | 1.44* [1.29, 1.61] | 0.97 [0.86, 1.10] |
|  | 3.36x10^-4^ | 0.7495 | 0.7759 | 0.7686 | 1.30x10^-10^ | 0.6809 |
| Prior MI | 1.79* [1.45, 2.17] | 1.18 [0.94, 1.46] | 0.88 [0.70, 1.10] | 0.85 [0.66, 1.08] | 2.05* [1.85, 2.25] | 1.39* [1.25, 1.54] |
|  | 1.41x10^-8^ | 0.1422 | 0.2770 | 0.1869 | 1.70x10^-47^ | 1.34x10^-9^ |
| Smoking | 1.27* [1.15, 1.41] | 1.26* [1.13, 1.40] | 0.96 [0.86, 1.08] | 1.02 [0.90, 1.15] | 1.33* [1.26, 1.40] | 1.24* [1.17, 1.31] |
|  | 4.58 x10^-6^ | 3.08x10^-5^ | 0.5348 | 0.7431 | 5.91x10^-26^ | 9.76x10^-15^ |

**Supplementary Table 4 caption:** **^**^**Comparison 1: COVID-19 positive (n=1,439) vs not COVID-19 positive (tested negative plus untested cohort) (n=494,838); Comparison 2: COVID-19 positive (n=1,439) vs COVID-19 test negative (n=5,660); Comparison 3: COVID-19 test negative (n=5,660) vs untested population (n=494,838). Results are odds ratio, 95% confidence interval, and p-value (from top to bottom) for each exposure. . For continuous variables (age, BMI) coefficients refer to the effect on odds of the outcome per five unit increase in the exposures, i.e. 5-year increase in age and 5kg/m^2^ increase in BMI. The remaining exposures are set as binary measures with results showing effect of change from non-disease to disease states, male sex vs female sex, BAME ethnicity vs White ethnicity; smoking history (current/previous) vs never smoked; ACEi use vs no ACEi use; ARB use vs no ARB use, on odds of the outcome. *indicates p-values <0.05. ACEi: Angiotensin Converting Enzyme inhibitor; ARB: Angiotensin Receptor Blocker; BMI: body mass index; coronavirus 2019: COVID-19; BAME: Black, Asian, and Minority ethnic; MI: myocardial infarction.
